# RNA‐binding protein DHX9 promotes glioma growth and tumor‐associated macrophages infiltration via TCF12

**DOI:** 10.1111/cns.14031

**Published:** 2022-11-15

**Authors:** Liang Liu, Xuelan Zhou, Shan Cheng, Yuyuan Ge, Baomin Chen, Jia Shi, Haoran Li, Suwen Li, Yongdong Li, Jiaqi Yuan, Anyi Wu, Xinglei Liu, Shilu Huang, Zhipeng Xu, Jun Dong

**Affiliations:** ^1^ Department of Neurosurgery Second Affiliated Hospital of Soochow University Suzhou China; ^2^ Department of Anesthesiology Second Affiliated Hospital of Soochow University Suzhou China; ^3^ Department of Neurosurgery Third Affiliated Hospital of Soochow University Changzhou China

**Keywords:** DHX9, glioma, macrophage infiltration, RNA‐binding protein

## Abstract

**Background:**

Glioma is the most common malignant tumor of the central nervous system, with high heterogeneity, strong invasiveness, high therapeutic resistance, and poor prognosis, comprehending a serious challenge in neuro‐oncology. Until now, the mechanisms underlying glioma progression have not been fully elucidated.

**Methods:**

The expression of DExH‐box helicase 9 (DHX9) in tissues and cells was detected by qRT‐PCR and western blot. EdU and transwell assays were conducted to assess the effect of DHX9 on proliferation, migration and invasion of glioma cells. Cocultured model was used to evaluate the role of DHX9 on macrophages recruitment and polarization. Animal study was performed to explore the role of DHX9 on macrophages recruitment and polarization in vivo. Bioinformatics analysis, dual‐luciferase reporter assay and chromatin immunoprecipitation (ChIP)‐qPCR assay was used to explore the relation between DHX9 and TCF12/CSF1.

**Results:**

DHX9 was elevated in gliomas, especially in glioblastoma multiforme (GBM). Besides promoting the proliferation, migration, and invasion of glioma cells, DHX9 facilitated the infiltration of macrophages into glioma tissues and polarization to M2‐like macrophages, known as tumor‐associated macrophages (TAMs). DHX9 silencing decreased the expression of colony‐stimulating factor 1 (CSF1), which partially restored the inhibitory effect on malignant progress of glioma and infiltration of TAMs caused by DHX9 knockdown by targeting the transcription factor 12 (TCF12). Moreover, TCF12 could directly bind to the promoter region of CSF1.

**Conclusion:**

DHX9/TCF12/CSF1 axis regulated the increases in the infiltration of TAMs to promote glioma progression and might be a novel potential target for future immune therapies against gliomas.

## Graphical abstract

DHX9/TCF12/CSF1 axis regulate the infiltration of TAMs to promote glioma progression and may be a novel potential target for future immune therapies against gliomas.

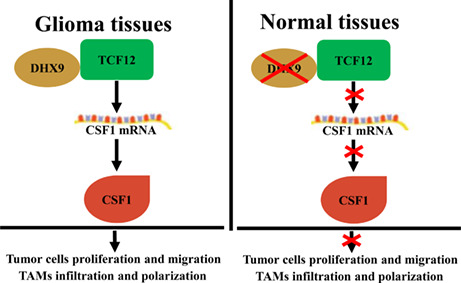

## 1 INTRODUCTION

Glioma is the most common malignant tumor of the central nervous system, with a strong invasive ability and high lethality, leading to great therapeutic failure.^1^, ^2^ Currently, the standard treatment for glioma is maximal safe resection, followed by radiotherapy and chemotherapy.^3^ Although some emerging treatments, such as immune therapies, have made certain progress in glioma treatment, the prognosis of glioma patients has not significantly improved.^4^ Considering the increasing application of individualized target therapies and immune therapies, molecular typing of both the tumor parenchyma and tumor microenvironment (TME) is crucial to guide glioma treatments. Therefore, clarifying the molecular mechanisms of both gliomagenesis and pro‐tumor effects of intra‐tumor immune cells is of great importance.

The TME is composed of various cellular components including tumor‐associated stromal and immune cells, comprehending the cytological basis for the high heterogeneity and refractoriness of gliomas.^5^, ^6^ These cells can trigger chronic inflammation and immunosuppression, hypoxia, and acidosis, and promote the development of glioma. The macrophages in the TME, known as tumor‐associated macrophages (TAMs), are the major cell subset in the stroma of gliomas, accounting for about 30–50% of total cells. Most TAMs are pro‐tumor M2 macrophages and contribute to tumor progression mainly by promoting angiogenesis, microenvironmental remodeling, ectopic growth, and immunosuppression.^7^, ^8^ Many studies have shown that TAMs are crucial to glioma progression and promoting the polarization from the M2 phenotype to M1 can reverse the malignant progression of glioma.^9^, ^10^ However, the underlying molecular mechanisms of TAMs polarization need further investigation before targeting them to glioma treatment.

The DExH‐box helicase 9 (DHX9) belongs to the DExH‐box family and is an NTP‐dependent RNA/DHA helicase. DHX9 was originally extracted from the bovine thymus but its homologous have been elucidated in humans, mice, and drosophila.^11^, ^12^ Human proteomic studies have found that DHX9 is widely expressed in various tissues, including the pancreas, skeletal muscle, and lymph nodes.^13^ Until now, DHX9 has been found to play important roles in maintaining cellular homeostasis and interacting with molecular chaperones to regulate DNA repair.^14^, ^15^ Previous studies have also indicated that DHX9 dysregulation is related to cancer progression.^15^, ^16^ However, its role in gliomas, especially in the polarization of TAMs, has not been fully clarified.

In the present study, we found that DHX9 was upregulated in gliomas, especially in GBMs. The functional experiments demonstrated that DHX9 promoted glioma progress and infiltration of TAMs into glioma tissues. The mechanistic investigations showed that DHX9 promote glioma progress by targeting the TCF12/CSF1 axis, indicating that DHX9 might be a novel target for precise immunotherapies against gliomas.

## 2 MATERIALS AND METHODS

### 2.1 Human clinical samples

Thirty glioma surgical specimens (10 cases of each grade: II, III, and IV) were collected at the Department of Neurosurgery, Second Affiliated Hospital of Soochow University. The corresponding small pieces of peritumor tissues (PTTs) were obtained during surgical exposure to intracerebral gliomas. Then, tissues were immediately immersed in an RNA protection solution and stored in liquid nitrogen (−80°C) for preservation. Informed consent was obtained from all patients. This study was approved by the Research Ethics Committee of the Second Affiliated Hospital of Soochow University.

### 2.2 Cell culture

Normal human astrocytes (NHAs), U251, A172, T98G, LN229, U118 and THP‐1 cells lines were purchased from Procell. NHAs and glioma cell lines were cultured in Dulbecco's modified Eagle medium (DMEM) supplemented with 10% fetal bovine serum (FBS, Sciencell). The THP‐1 cells were cultured in RPMI 1640 medium with 10% heat‐inactivity FBS. Besides, 0.1 μg/ml phorbol‐12‐myristate‐13‐acetate (PMA, Sigma Aldrich) was used to induce differentiation of THP‐1 cells into macrophages. All cells were placed in an incubator with an atmosphere of 5% CO_2_ at 37°C.

### 2.3 Plasmid construction and transfection

Small interfering RNAs (siRNAs) targeting DHX9, TCF12, and CSF1 and their corresponding negative controls were purchased from GenePharma. The transfection and selection were conducted according to the manufacturer's protocol. Coding sequences of DHX9, TCF12, and CSF1 were amplified with the corresponding primers by PCR. Next, the PCR products were inserted into a pcDNA(+) Vector constructed based on an overexpression plasmid. Then, the plasmids were transfected into cells with Lipofectamine 3000 (Invitrogen).

### 2.4 RNA extraction and quantitative real‐time PCR (qRT‐PCR)

Total RNA was extracted from tissues and cells with TRIzol (Sigma Aldrich). After concentration and quality control, total RNA was reversely transcribed into cDNAs with a reverse transcription cDNA Kit (Thermo Fisher). Next, qRT‐PCR was conducted with a Real‐Time PCR Kit (Takara) and the ABI 7500 system (Applied Biosystems). GAPDH was used as the internal control. The primers used in this study were shown below: DHX9, forward: 5′‐AGC TGT GGC TAC AGC GTT CGAT‐3′; reverse: 5′‐CTG ATT CCT CGA ATG CCT GCTTC‐3′; TCF12, forward: 5′‐CCA GCA GTT CAC CTT ACG TTGC‐3′; reverse: 5′‐GCC TTT CCA AGT GCA TCA CCTG‐3′; CSF1: forward: 5′‐AGC TGT GGC TAC AGC GTT CGAT‐3′; reverse: 5′‐GCA ATC AGG CTT GGT CAC CACA‐3′; GAPDH: forward: 5′‐GTC TCC TCT GAC TTC AAC AGCG‐3′; reverse: 5′‐ACC ACC CTG TTG CTG TAG CCAA‐3′.

### 2.5 Western blot

Total protein was extracted from tissues and cells with the RIPA lysis buffer (Beyotime). After determining the concentration using the Enhanced BCA Protein Assay Kit (Beyotime), total protein was separated by sodium dodecyl sulfate‐polyacrylamide gel electrophoresis (SDS‐PAGE) and transferred to a polyvinylidene difluoride membrane (Millipore). After incubation with 5% non‐fat milk for 2 h, the membrane was successively incubated with the corresponding primary and secondary antibodies. Finally, chemiluminescence visualization was performed with Tanon 4600SF (Tanon). The primary antibodies used in this study were: mouse anti‐DHX9 (Proteintech), rabbit anti‐TCF12 (Proteintech), rabbit anti‐CSF1 (Zen BioScience), and rabbit anti‐GAPDH (Zen BioScience).

### 2.6 Cell counting kit (CCK‐8) assay

First, 2 × 10^5^ TAMs per well were seeded in 24‐well plates. After coculturing with glioma cells for 48 h, 20 μl of the CCK‐8 solution was added to each well, followed by incubation for 4 h. Finally, the absorbance at 450 nm was measured with a microplate reader (BioTek).

### 2.7 5‐Ethynyl‐20‐deoxyuridine (EdU) assay

3 × 10^4^ cells per well were seeded in 96‐well plates overnight. After treatment with EdU (RiboBio) solution for 2 h, cells were fixed with 4% paraformaldehyde for 15 min and permeabilized with 0.1% TritonX‐100 for 30 min. Next, cells were incubated with Apollo dye solution (RiboBio) for 30 min and DAPI for 3 min in the dark. Finally, EdU‐positive cells were detected with a fluorescence microscope (Olympus).

### 2.8 Transwell assay

Transwell inserts (8.0 μm, Corning) were used to evaluate the migration and invasion ability of glioma cells. Briefly, 4 × 10^4^ cells suspended in FBS‐free DMEM were added to the upper chambers and 600 μl of 10% FBS‐contained DMEM was added to the low chambers. After 48 h, cells in the upper chamber were wiped and the cells that migrated or invaded were captured with a microscope (Olympus). Matrigel (BD) was precoated on the upper chamber for the invasion assay.

### 2.9 Immunohistochemistry (IHC) assay

Sections were prepared with tissues embedded in paraffin and boiled in sodium citrate. Next, sections were blocked with goat serum and incubated with primary antibodies overnight at 4°C. Then, horseradish peroxidase‐conjugated secondary antibody and 3′‐diaminobenzidine were applied for visualization, followed by counterstaining with hematoxylin. Finally, sections were analyzed under a microscope (Olympus).

### 2.10 Enzyme‐linked immunosorbent assay (ELISA)

The concentration of CSF1 in supernatants was measured with a CSF1 enzyme‐linked immunosorbent assay kit (Invitrogen), according to the manufacturer's protocol. First, the supernatant was added to a 96‐well plate and incubated for 3 h, followed by successive incubation with biotinylated primary antibody for 2 h and streptavidin‐HRP solution for 45 min. Next, the TMB development solution was added to each well and incubated for 10 min. Finally, absorbance at 450 nm was measured with a microplate reader (BioTek).

### 2.11 Dual‐luciferase reporter assay

The CSF1 promoter region was amplified and cloned into a luciferase reporter vector (Promega). Site‐directed mutagenesis was performed to construct CSF1‐MUT. CSF1‐WT (wild type) was used as a negative control. Then, the Renilla luciferase signal and luciferase value were determined with a dual‐luciferase system (Promega).

### 2.12 Chromatin immunoprecipitation (ChIP)‐qPCR assay

Cells overexpressing DHX9 were cross‐linked with formaldehyde, lysed in SDS buffer, and mechanically sheared by sonication to fragment DNA. Protein‐DNA complexes were precipitated using IgG and anti‐TCF12 antibody. After that, the eluted DNA fragment was detected with the primers specific for CSF1 promoter and SYBR premix.

### 2.13 Experiments in vivo

Procedures were approved by the Institutional Animal Care and Use Committee of the Soochow University. First, 12 (6 per group) Balb/c female nude mice (4 weeks) were purchased from Vital River (Beijing, China). Luciferase‐expressing LN229 cells stably transfected with sh‐DHX9 or negative control were injected into the right caudate nucleus with stereotaxic techniques. Then, dynamic observation of the growth of intracerebral xenografts was conducted with a bioluminescence imaging system (IVIS Lumina II) and Living Image software (Caliper Life Science).

### 2.14 Statistical analyses

All statistical analyses were conducted with GraphPad Prism 9.0. Results are presented as means ± SD based on three independent experiments. The normality of the data distribution was analyzed by the Shapiro–Wilk test. Statistical comparisons between two groups were conducted by the two‐tailed Student's test, and comparisons between more than two groups were analyzed by Analysis of Variance (ANOVA). A *p* < 0.05 (*) was considered statistically significant.

## 3 RESULTS

### 3.1 DHX9 increases and is correlated with glioma grades

**FIGURE 1:**
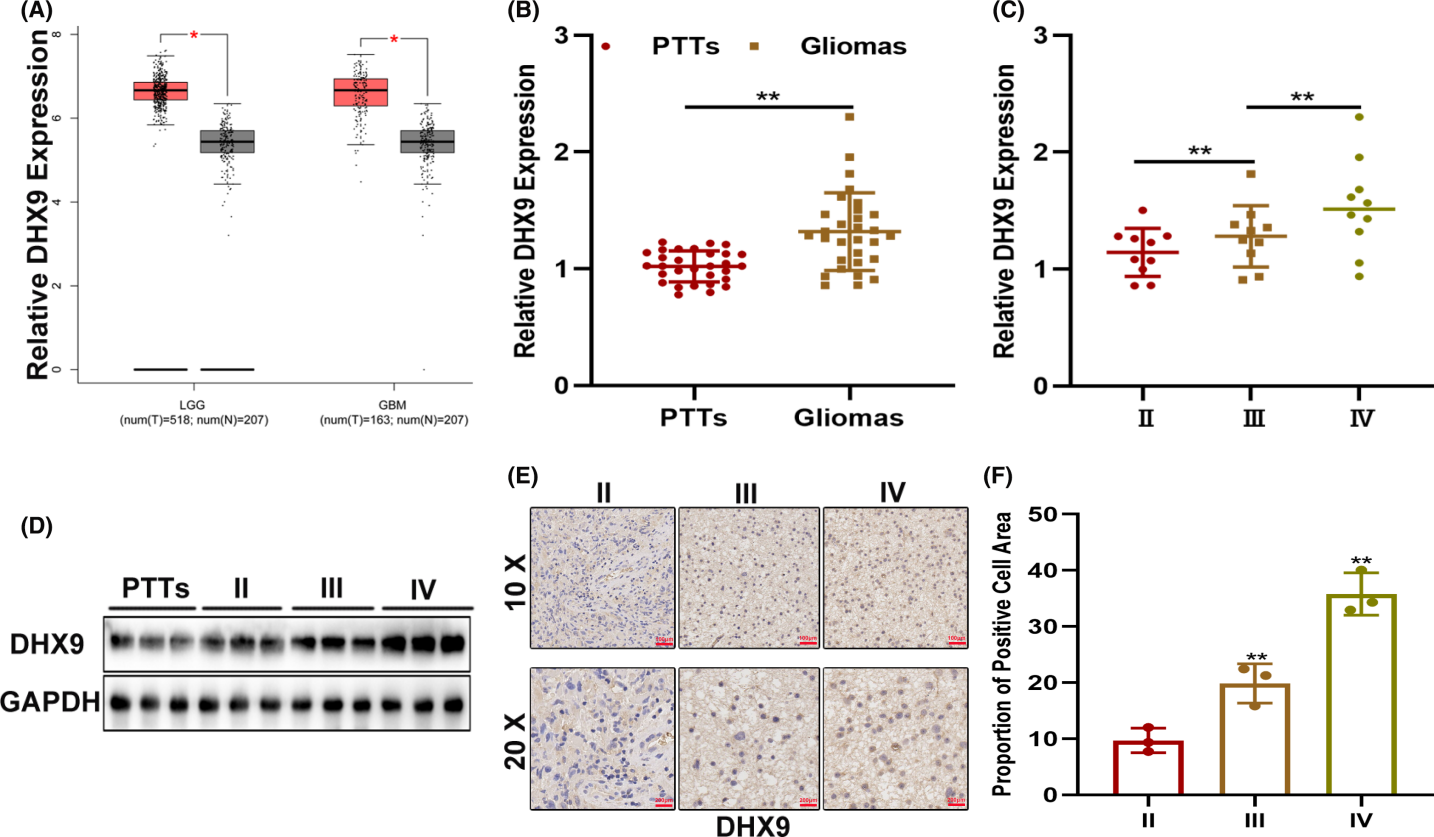
DHX9 increases and is correlated with glioma grades. (A) DHX9 is elevated in gliomas as indicated by the GEPIA public database. (B) DHX9 is upregulated in clinical samples of glioma. (C, D) Levels of DHX9 in different glioma grades detected by qRT‐PCR and Western blot. (E, F) Levels of DHX9 in different glioma grades detected by IHC.

The expression profile of DHX9 in gliomas was analyzed using GEPIA. The data showed that DHX9 was higher expressed in gliomas than in normal brain tissues (Figure 1A). Expression pattern of DHX9 in gliomas based on TCGA was shown in Figure S1. To further validate this expression profile, the expression of DHX9 was verified in clinical samples by qRT‐PCR and Western blot. Compared to PTTs, the levels of DHX9 were higher in gliomas, especially in GBMs (Figure 1B–D). Additionally, the relation between DHX9 levels and the grades of gliomas was further confirmed by IHC (Figure 1E,F). Altogether, these results indicated that DHX9 is a promising biomarker for gliomas.

### 3.2 DHX9 knockdown suppresses the malignant phenotype of gliomas in vitro

**FIGURE 2:**
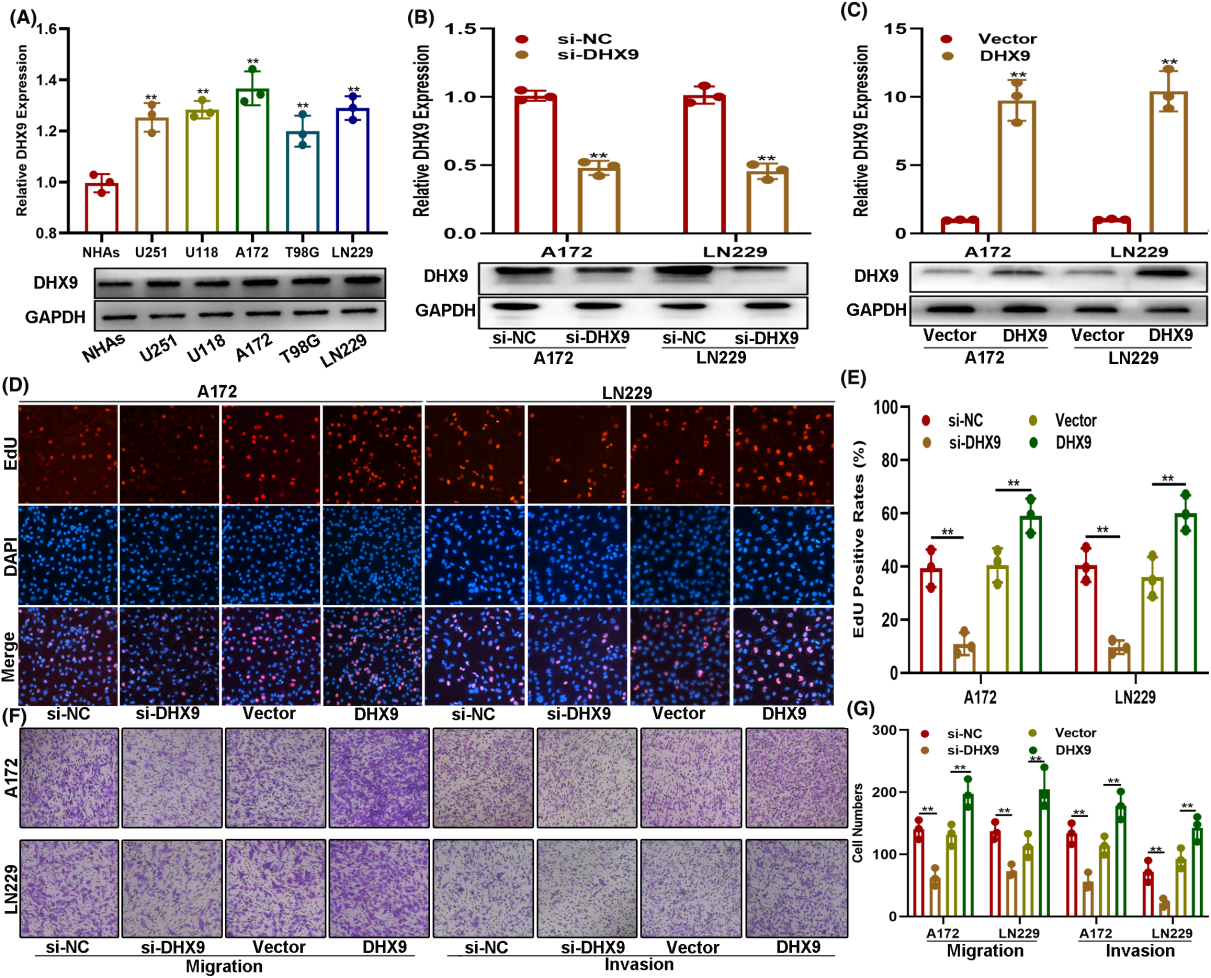
DHX9 knockdown suppresses the malignant phenotype of gliomas in vitro. (A) Levels of DHX9 in NHAs and glioma cells by qRT‐PCR and Western blot. (B, C) The transfection efficiency was verified by qRT‐PCR and Western blot. (D, E) EdU assay of cells transfected with si‐NC, si‐DHX9, vector, or DHX9‐overexpression plasmids. (F, G) Transwell assay of cells transfected with si‐NC, si‐DHX9, vector, or DHX9‐overexpression plasmids

To investigate the function of DHX9 on glioma progress, functional assays were performed with human glioma cell lines. First, the levels of DHX9 were analyzed in these cells. The qRT‐PCR and Western blot assays showed that, compared with NHAs, DHX9 was higher expressed in glioma cells, particularly in A172 and LN229 cell lines (Figure 2A). Next, DHX9‐downregulated or overexpressed A172 and LN229 cells were constructed by plasmid transfection. The transfection efficiency was verified by qRT‐PCR and Western blot (Figure 2B,C). The EdU assays indicated that DHX9 silencing impaired the proliferation of glioma cells, whereas DHX9 overexpression accelerated cell growth (Figure 2D,E). The Transwell assays showed that DHX9 knockdown suppressed the migration and invasion of glioma cells, while DHX9 overexpression led to opposite effects (Figure 2F,G). These findings indicated that DHX9 knockdown can inhibit glioma's malignant phenotype in vitro.

### 3.3 DHX9 increased the recruitment of TAMs in vitro

**FIGURE 3:**
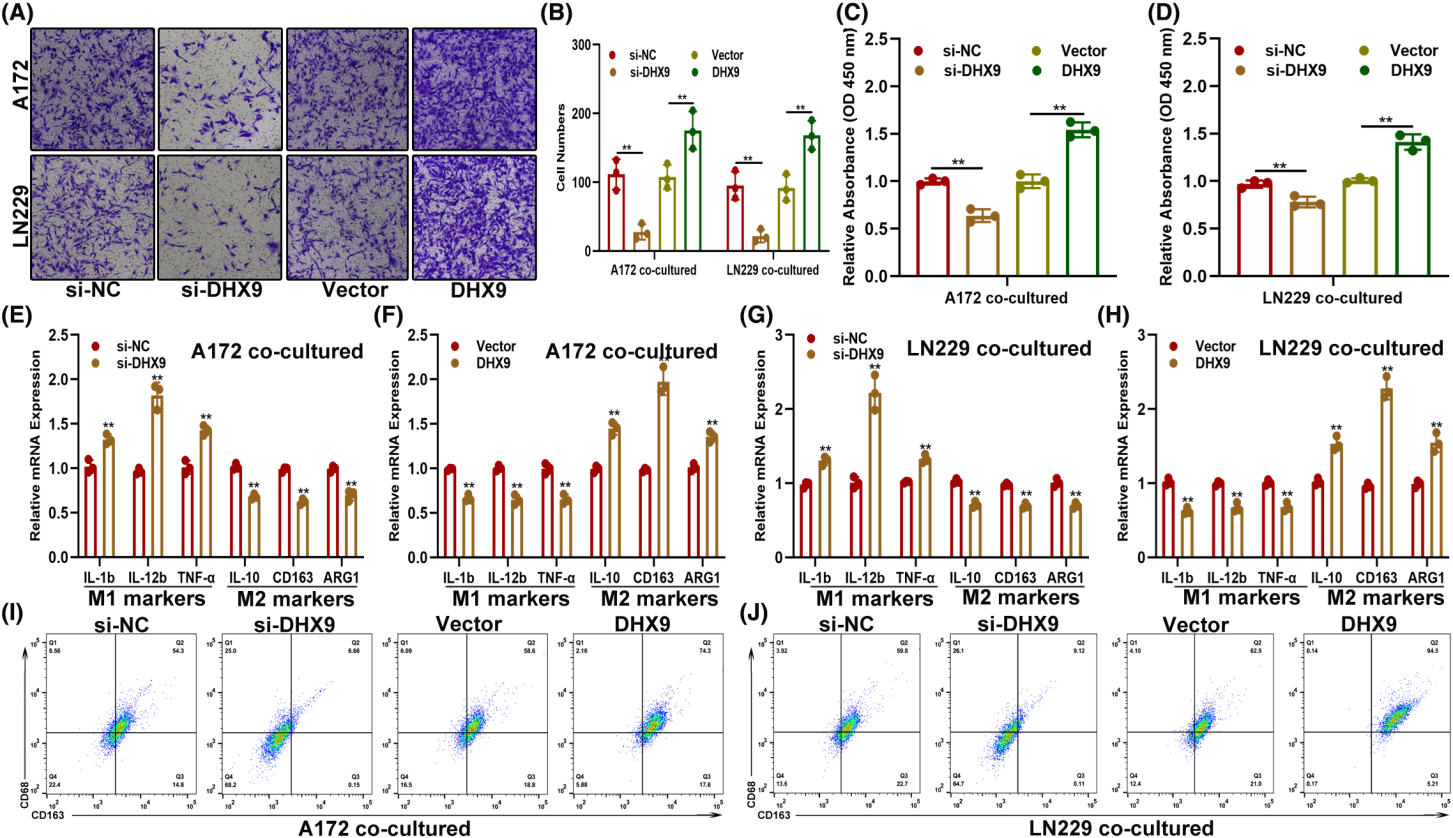
DHX9 increases the recruitment of TAMs in vitro. (A, B) Recruitment of TAMs by glioma cells transfected with si‐NC, si‐DHX9, vector, or DHX9‐overexpression plasmids. (C, D) Proliferation ability of TAMs cocultured with indicated glioma cells. (E–H) Expression of markers associated with M1 and M2 polarization of TAMs cocultured with indicated glioma cells by qRT‐PCR. (I, J) The ratio of M2 macrophages (CD68^+^/CD163^+^) in indicated groups was detected by flow cytometry

TAMs are the main immune cells in the glioma microenvironment, accounting for 30–50% of total tumor cells, and are closely related to glioma progression.^17^, ^18^ To explore whether DHX9 was involved in the infiltration of TAMs in the glioma microenvironment, an in vitro coculture model was constructed based on Transwell chambers. The Transwell migration assay showed that, compared to the control group, DHX9‐overexpressed glioma cells promoted the migration of PMA‐induced THP‐1 cells, while DHX9 silencing weakened this process (Figure 3A,B). The CCK‐8 assay showed that macrophages derived from PMA‐induced THP‐1 cells presented enhanced proliferative ability when cocultured with DHX9‐overexpressed glioma cells (Figure 3C,D). Furthermore, the expression of M1 or M2 subtype markers in cocultured macrophages was detected by qRT‐PCR. Results demonstrated that, when cocultured with DHX9‐overexpressed glioma cells, macrophages exhibited decreased expression of M1 markers (IL‐1b, IL‐12b, and TNF‐α) and increased expression of M2 markers (IL‐10, CD163, and ARG1). Meanwhile, when cocultured with DHX9‐silenced glioma cells, the macrophages presented an opposite trend (Figure 3 E–H). In addition, flow cytometry analysis presented that when cocultured with DHX9‐overexpressed glioma cells, the ratio of CD68^+^/CD163^+^ cells were significantly higher than the control (Figure 3I,J). Altogether, these results indicated that DHX9 contributes to the infiltration and M2 polarization of TAMs.

### 3.4 DHX9 knockdown suppressed glioma growth and macrophage infiltration in vivo

**FIGURE 4:**
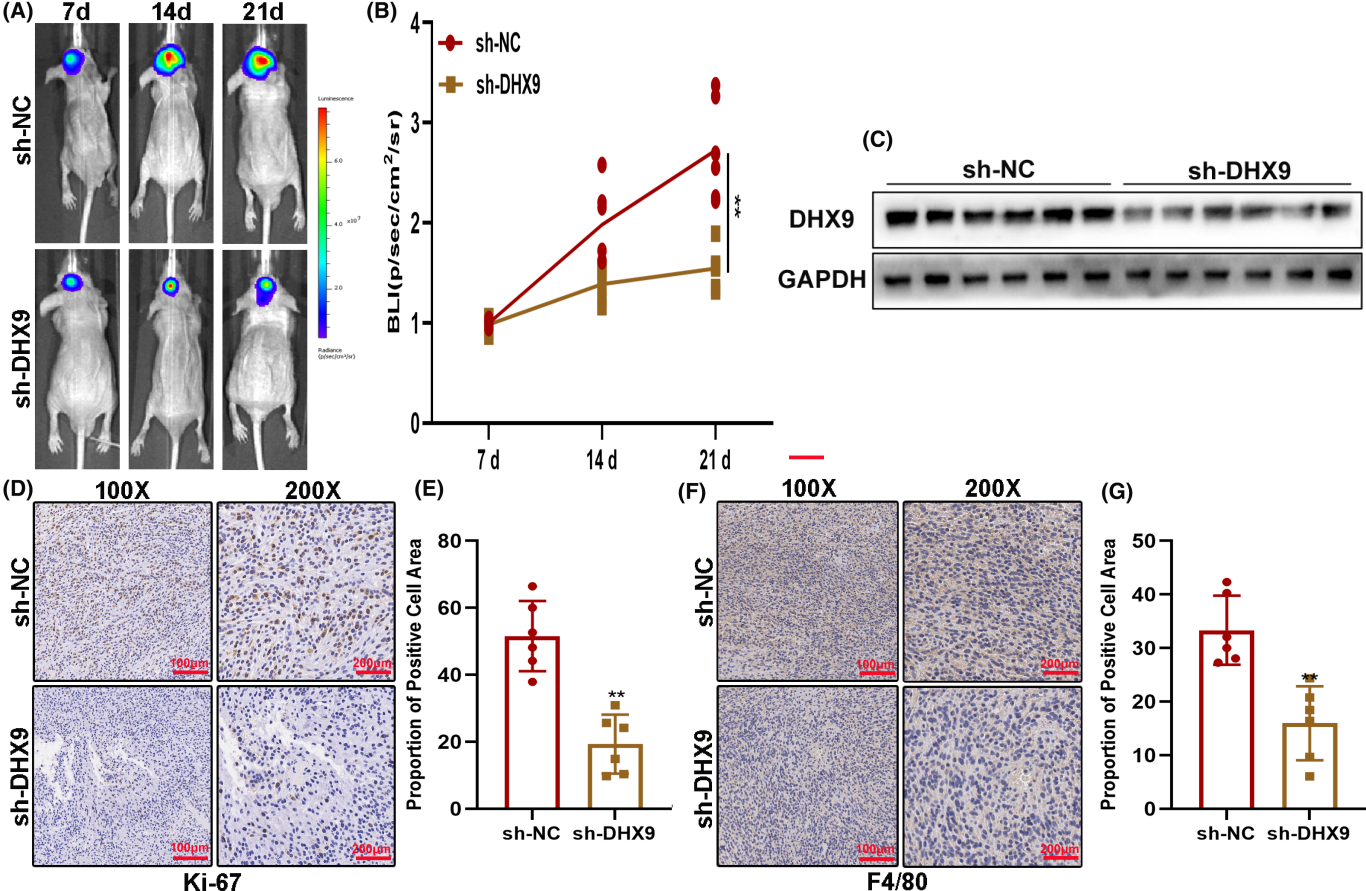
DHX9 knockdown suppressed glioma growth and macrophage infiltration in vivo. (A, B) The in vivo assay showed that sh‐DHX9 inhibits tumor growth. (C) Levels of DHX9 in the tumors formed in nude mice by Western blot. (D, E) Ki‐67‐positive cells in the tumors by ki‐67 staining. (F, G) F4/80‐positive cells in the tumors formed in nude mice by IHC

The imagining analysis was carried out to evaluate the role of DHX9 on glioma growth in vivo. The results showed that inhibition of DHX9 suppressed the intracranial growth of glioma (Figure 4A,B). Then, the tumors formed were removed after tumor‐bearing mice were sacrificed. The Western blot showed that DHX9 was downregulated in DHX9‐knockdown gliomas (Figure 4C). The Ki‐67 staining of tumor tissue sections indicated that ki‐67 positive‐stained cells decreased in the DHX9 knockdown groups (Figure 4D,E). Furthermore, the IHC assay showed that F4/80 positive cells significantly decreased in the DHX9 knockdown groups compared to controls (Figure 4F,G). Altogether, these data suggested that DHX9 knockdown suppressed glioma growth and macrophage infiltration in vivo.

### 3.5 CSF1 is involved in DHX9‐mediated malignant phenotypes and infiltration of TAMs in gliomas

**FIGURE 5:**
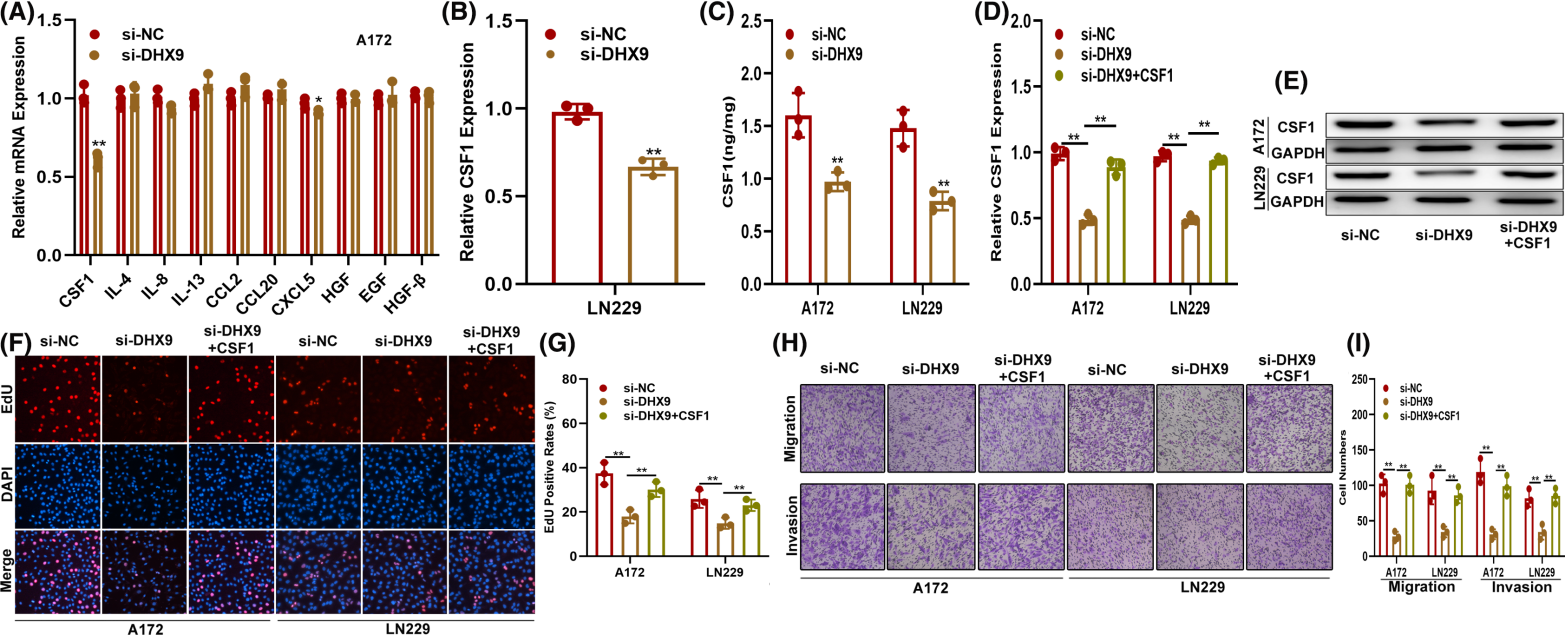
CSF1 is involved in the DHX9‐mediated malignant phenotype of gliomas. (A) Level of TAMs recruitment‐associated cytokines in DHX9 silenced A172, which detected by qRT‐PCR. (B) Level of CSF1 in LN229 cells transfected with si‐NC or si‐DHX9 by qRT‐PCR. (C) Level of CSF1 in the supernatants of A172 and LN229 cells transfected with si‐NC or si‐DHX9 by ELISA. (D, E) Levels of CSF1 in glioma cells transfected with si‐NC, si‐DHX9, or si‐DHX9 with CSF1‐overexpression plasmids by qRT‐PCR and Western blot. (F, G) EdU assay of glioma cells transfected with si‐NC, si‐DHX9, or si‐DHX9 with CSF1‐overexpression plasmids. (H, I) Migration and invasion ability of the indicated cells by the Transwell assay

**FIGURE 6:**
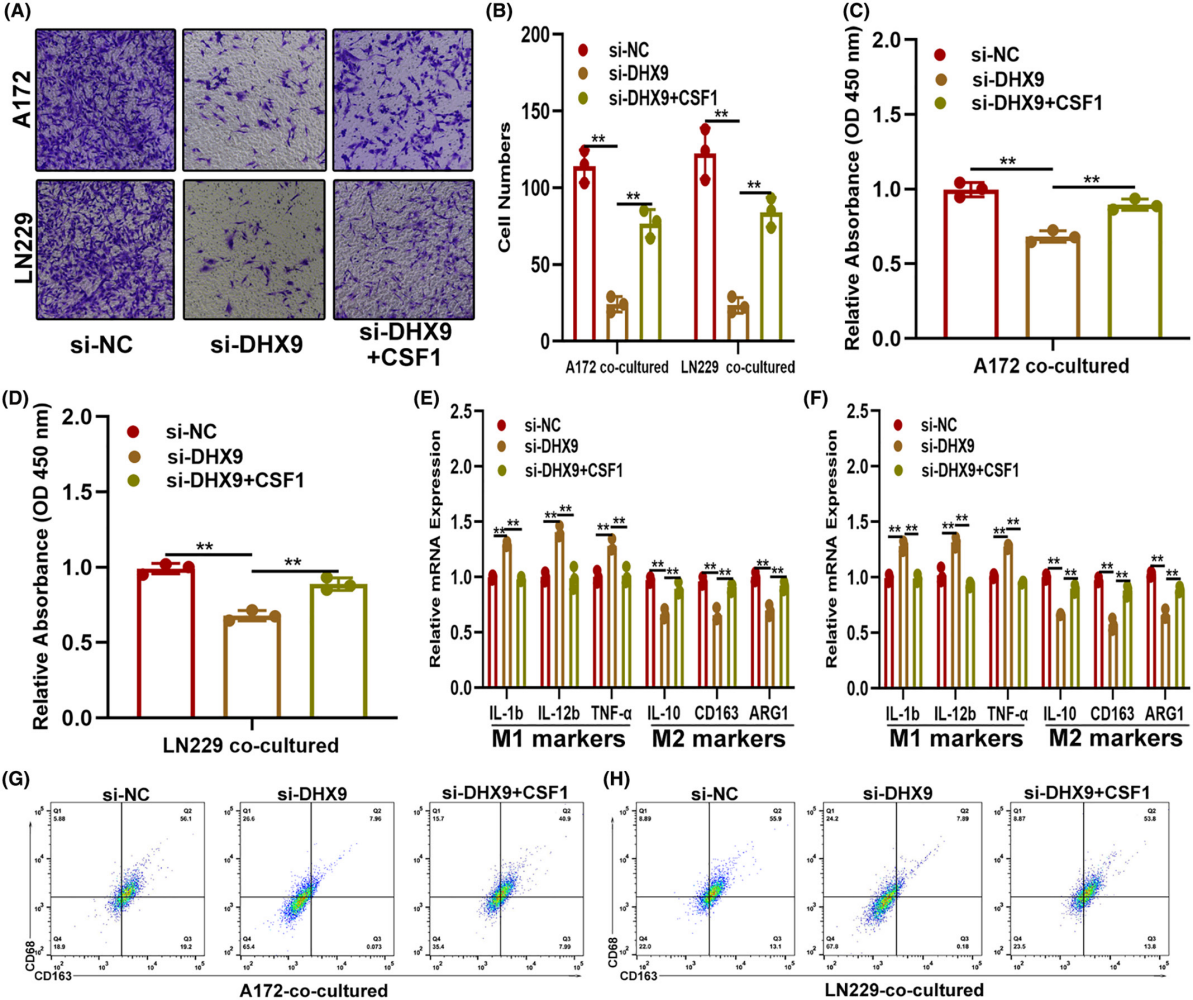
CSF1 is involved in the DHX9‐mediated infiltration of TAMs in glioma tissues. (A, B) Coculture model of the TAMs recruited by the indicated glioma cells. (C, D) Proliferation ability of TAMs recruited by indicated glioma cells by the CCK‐8 assay. (E, F) Expression of markers associated with M1 and M2 polarization of TAMs recruited by indicated glioma cells by qRT‐PCR. (G, H) The ratio of M2 macrophages (CD68^+^/CD163^+^) in indicated groups was detected by flow cytometry

During in‐depth studies of the tumor microenvironment, TAMs are described as closely related to the occurrence and development of tumors.^19^, ^20^ Meanwhile, the infiltration of TAMs is also related to chemokines, such as TGF‐β, IL‐4, IL‐8, and CSF1.^21^, ^22^ Based on these theories, we explored whether DHX9 promoted glioma progression and infiltration of TAMs via regulating the expression of chemokines. The mRNA level of TAMs infiltration‐associated chemokines in DHX9‐silenced A172 cells was detected by qRT‐PCR and demonstrated that CSF1 decreased most obviously (Figure 5A). CSF1 was decreased in si‐DHX9 transfected LN229 as well (Figure 5B). More interesting, CSF1 decrease was also observed in the supernatants of DHX9‐silenced A172 and LN229 cells (Figure 5C). Next, to investigate the role of CSF1 in DHX9‐mediated glioma progression and TAMs infiltration, A172 and LN229 cells were transfected with NC, si‐DHX9 alone, or si‐DHX9 with CSF1‐overexpression plasmids. The levels of CSF1 in these cells were detected by qRT‐PCR and Western blot. Results showed that si‐DHX9 decreased the levels of CSF1, while CSF1 overexpression could reverse this effect (Figure 5D,E). Moreover, The EdU assay showed that CSF1 overexpression abolished the proliferation inhibition caused by si‐DHX9 (Figure 5F,G). Similarly, the Transwell assay indicated that CSF1 overexpression restored the effect of si‐DHX9‐induced migration and invasion suppression in both A172 and LN229 cells (Figure 5H,I). Meanwhile, the Transwell coculture assay showed that CSF1 overexpression could partly overcome the reduction of macrophages recruitment caused by DHX9 silencing (Figure 6A,B). Simultaneously, compared to TAMs cocultured with DHX9‐silenced glioma cells, the proliferative capacity of TAMs was higher in si‐DHX9 with CSF1‐overexpression plasmid‐transfected glioma cells (Figure 6C,D). Additionally, the expression of markers associated with the classification of TAMs showed that si‐DHX9 directed the polarization of macrophages towards the M1 subtype, while this effect could be partly reversed by CSF1 overexpression (Figure 6 E,F). Flow cytometry analysis presented that when cocultured with si‐DHX9 glioma cells, the ratio of CD68^+^/CD163^+^ cells were significantly lower than the control; however, this effect could be partly reversed by cocultured with CSF1 overexpressed glioma cells (Figure 6G,H). These data indicated that DHX9 promoted the glioma malignant phenotype and infiltration of TAMs by regulating CSF1.

### 3.6 DHX9 mediated the expression and function of CSF1 via TCF12

**FIGURE 7:**
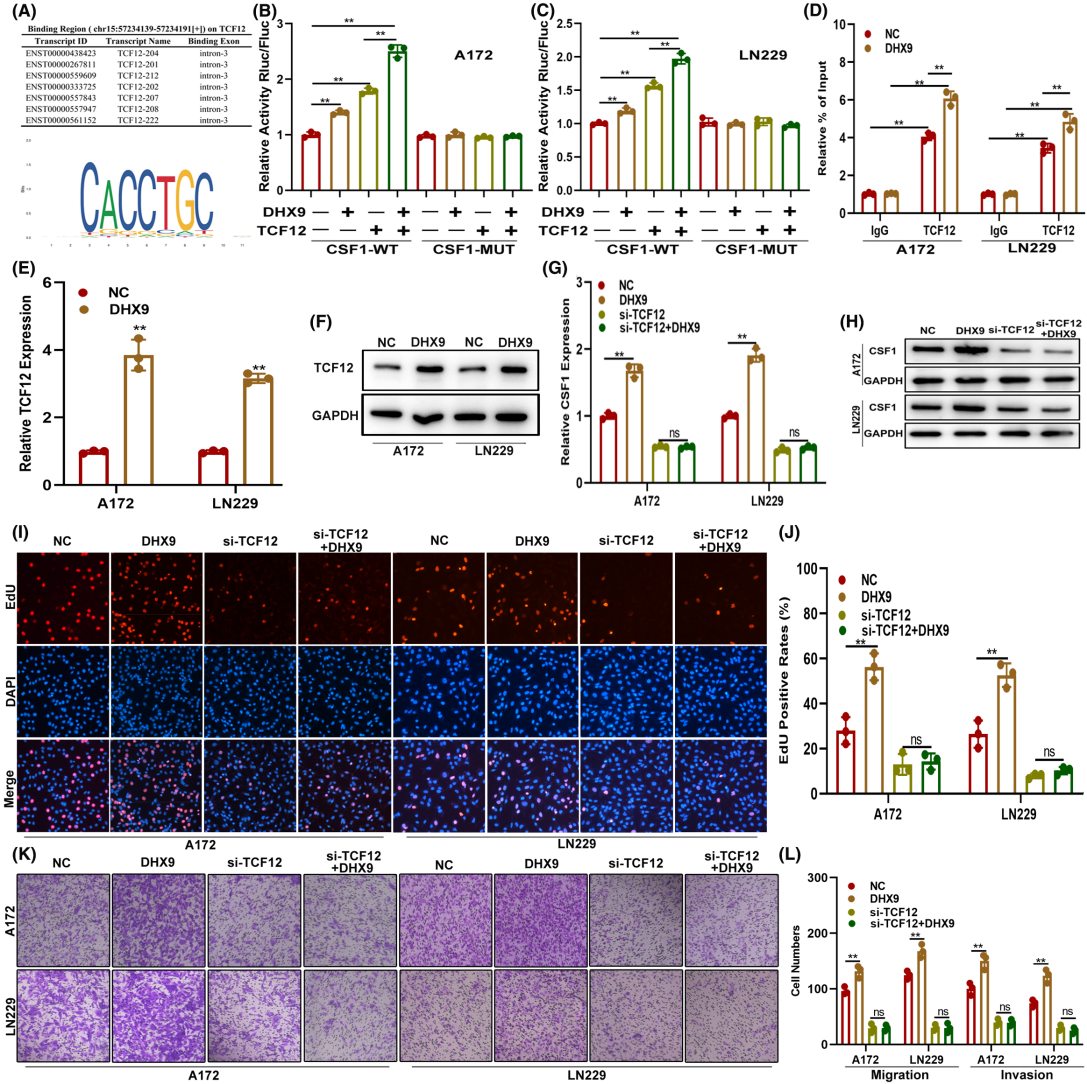
DHX9 mediates the expression and function of CSF1 on glioma's malignant phenotype by targeting TCF12. (A) Data from starBase, UCSC and JASPAR indicating that TCF12 could bind to DHX9 and CSF1. (B, C) Luciferase reporter assay was used to validate the interaction between DHX9 and CSF1. (D) ChIP‐qPCR assay showed that DHX9 facilitated the binding of TCF12 to the CSF1 promoter. (E, F) TCF12 was upregulated in DHX9‐overexpressed A172 and LN229, which detected by qRT‐PCR and Western blot. (G, H) Levels of CSF1 in glioma cells transfected with NC, DHX9, si‐TCF12, or si‐TCF12 together with CSF1‐overexpression plasmids by qRT‐PCR and Western blot. (I, J) EdU assay of glioma cells transfected with NC, DHX9‐overexpression, si‐TCF12, or si‐TCF12 with DHX9‐overexpression plasmids. (K, L) Migration and invasion ability of the indicated cells by the Transwell assay

**FIGURE 8:**
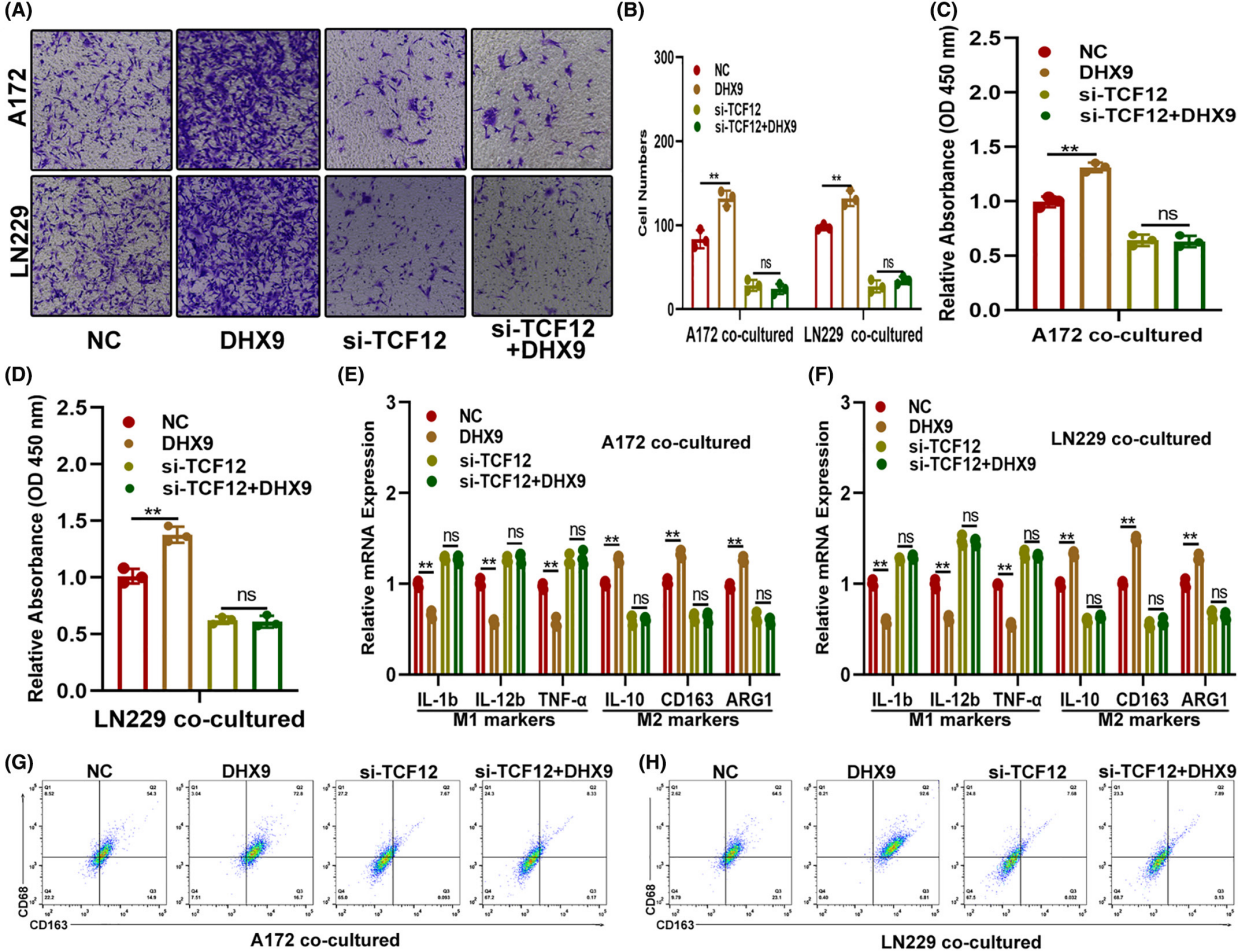
DHX9 mediates the expression and function of CSF1 on TAMs infiltration by targeting TCF12. (A, B) The coculture model shows the TAMs recruited by indicated glioma cells. (C, D) The proliferation ability of TAMs recruited by indicated glioma cells by the CCK‐8 assay. (E, F) Expression of markers associated with M1 and M2 polarization of TAMs recruited by indicated glioma cells by qRT‐PCR. (G, H) The ratio of M2 macrophages (CD68^+^/CD163^+^) in indicated groups was detected by flow cytometry

To further investigate the role of DHX9 on CSF1 expression and function, public databases were carefully searched. However, no evidence that could prove that DHX9 could regulate CSF1 directly, which indicated the possibility that an intermediary regulator may combined them. By searching the starBase, UCSC and JASPAR databases, TCF12 was selected as a potential candidate that could simultaneously bind to DHX9 and CSF1 (Figure 7A). To validate the relation between TCF12 and DHX9/CSF1, wild type (WT) and a TCF12‐binding motif mutated (MUT) CSF1 promoter were applied to construct a luciferase reporter. The luciferase reporter assay documented that upregulation of DHX9 significantly increased the CSF1 promoter‐driven luciferase activity in the WT group rather than the MUT group (Figure 7B, C) and ChIP‐qPCR assay showed that DHX9 facilitated the binding of TCF12 to the CSF1 promoter (Figure 7D). Moreover, TCF12 was upregulated in DHX9‐overexpressed A172 and LN229 both mRNA and protein level (Figure 7 E,F). To investigate whether DHX9 regulated the function of CSF1 on glioma in a TCF12‐dependent manner, glioma cells were genetically modified by transfecting NC, DHX9‐overexpression plasmids, si‐TCF12, or si‐TCF12 plus DHX9‐overexpression plasmid. Then, the expression of CSF1 in these cells was assessed by qRT‐PCR, which showed that the DHX9‐overexpression plasmid failed to upregulate CSF1 in si‐TCF12‐transfected glioma cells (Figure 7G,H). The cell function assay showed that TCF12 silencing diminished the promotion effect on glioma proliferation, migration and invasion induced by DHX9 (Figure 7I–L). Furthermore, TCF12 knockdown abolished DHX9‐mediated recruitment, proliferation and polarization of macrophages towards the M2 phenotype (Figure 8). These results demonstrated that DHX9 mediated the expression and function of CSF1 on glioma's malignant phenotype and infiltration of TAMs via TCF12.

## 4 DISCUSSION

Glioma is a tumor with poor prognoses (short survival and high mortality). Although the understanding of glioma tumorigenesis has progressed in recent years, the survival of glioma patients has not significantly improved compared to other tumors.^1^, ^23^ Until now, the role of the immune microenvironment in the progression of gliomas has gradually become a research hotspot. Increasing studies have shown that remodeling of the immunosuppressive microenvironment is closely related to glioma progression and that TAMs in this environment are closely related to immune escape and therapeutic challenges in glioma patients.^24^, ^25^ Hence, revealing tumor immune regulatory mechanisms and key regulatory molecules in the glioma immune microenvironment is of great significance for the development of novel TAM‐targeted therapies for glioma.

The DHX9 gene is located in chromosome 1 and participates in multiple cell activity processes. It also maintains gene stability by regulating gene expression at different stages and degrading abnormal nucleotides to ensure the fidelity and efficiency of DNA replication. The abnormal function of DHX9 can lead to the occurrence of many diseases, including tumors. Previous studies have shown that DHX9 is essential for the survival of tumor cells, and inhibiting DHX9 results in fatal damage to tumor cells without affecting the function of normal cells.^13^ Additionally, the downregulation of DHX9 can suppress the progression of certain tumors, such as lung adenocarcinoma.^26^ Until now, the expression and function of DHX9 in glioma have not been fully investigated. Yi et al. observed that DHX9 decreased in U87 cells treated with temozolomide (TMZ) for a week,^27^ but the underlying mechanisms were not clarified. In the present study, we found that DHX9 was upregulated in gliomas, especially in GBMs. Furthermore, we demonstrated that DHX9 silencing can inhibit the proliferation, migration, and invasion of glioma cells in vitro and tumor growth in vivo. Besides, DHX9 can promote the infiltration of TAMs in the glioma microenvironment. These findings indicated that DHX9 contributed to the maintenance of both the malignant phenotype and the formation of the immunosuppressive microenvironment of gliomas.

Increasing evidence has proved that chemokines and cytokines are crucial for both tumor growth and recruitment of TAMs.^28^, ^29^ Thus, to reveal the mechanisms by which DHX9 mediates the malignant progression of gliomas, we analyzed the expression of major chemokines in DHX9 silenced glioma cells. Our results showed that CSF1 was significantly downregulated in DHX9‐downregulated A172 and LN229. In addition to stimulating the accumulation of macrophages in tumors, CSF1 can also promote the survival and differentiation of monocytes into macrophages, increasing the infiltration of macrophages in tumor tissues, thereby promoting tumor metastasis and angiogenesis. Xin et al showed that CSF1 can induce prostate intraepithelial neoplasia by promoting the infiltration of immune cells and regulating the polarization of macrophages.^30^ Moreover, Guan et al. reported that CSF1 was regulated by FOXO1 and induced the polarization of TAMs from the M0 towards the M2 phenotype, promoting the progression of esophageal squamous cell carcinoma.^31^ Some studies also reported that CSF1 promotes tumor growth and infiltration of TAMs in gliomas;^32^, ^33^ however, the regulatory network remained unclear. In the current study, we showed that CSF1 can abolish the inhibitory effect of DHX9 silencing behind the proliferation, migration, and invasion of glioma cells and infiltration of TAMs in vivo, which implied that CSF1 was the functional target of DHX9.

However, the data from genetic research‐related databases indicated that there is no direct targeting between DHX9 and CSF1, indicating the necessity of an intermediary to form a complete signaling pathway. DHX9 is an RNA‐binding protein that can directly bind to downstream target proteins. After bioinformatics retrieval and prediction, TCF12 was found not only to be harbored by DHX9 but also binding to the promoter of CSF1. TCF12 is a transcription factor (TF) that can bind to various gene promoters and regulate processes such as embryonic development. Moreover, TCF12 has been proved as an oncogene in different tumors, including pancreatic carcinoma,^34^ melanoma,^35^ and hepatocellular carcinoma.^36^ Consistent with previous findings,^37^ we found that TCF12 silence could inhibit the malignant phenotype of gliomas. Furthermore, TCF12 knockdown eliminated the carcinogenic effect caused by DHX9 overexpression, suggesting that DHX9 promoted the CSF1‐induced glioma growth and recruitment of TAMs in a TCF12‐dependent manner.

## 5 CONCLUSION

In conclusion, we demonstrated that DHX9 was elevated in gliomas and promoted the proliferation, migration, invasion of glioma cells, and the infiltration of TAMs by targeting the TCF12/CSF1 axis. Our current results suggested that DHX9 can be a potential immune‐related therapeutic target against gliomas.

## AUTHOR CONTRIBUTIONS

LL, XZ, SC, and YG visualization, writing original draft, investigation. JS, SL, YL, and JY formal analysis and data curation. AW, LH, and YG resources. XL, BC, and SH validation, software. JD supervision, project administration, draft review and editing, and funding acquisition.

## FUNDING INFORMATION

This research was funded by Natural Science Foundation of Jiangsu Province, China (No. BK20201172), Key project of Jiangsu Health Commission (ZDB2020016), Jiangsu province key research and development program: Social development project (BE2021653), Changzhou Sci & Tech Program (CJ20210066), Young Talent Development Plan of Changzhou Health Commission (2020‐233‐CZQM2020013).

## CONFLICT OF INTEREST

The authors declare no conflict of interest.

## Supporting information


Figure S1

## Data Availability

The data that support the findings of this study are available from the corresponding author upon reasonable request.
