# Supplementary figures and images for: RNA‐binding protein DHX9 promotes glioma growth and tumor‐associated macrophages infiltration via TCF12
Source: CNS Neurosci Ther. 2022 Nov 15;29(4):988–99. doi: 10.1111/cns.14031 (PMC10018109; doi:10.1111/cns.14031)

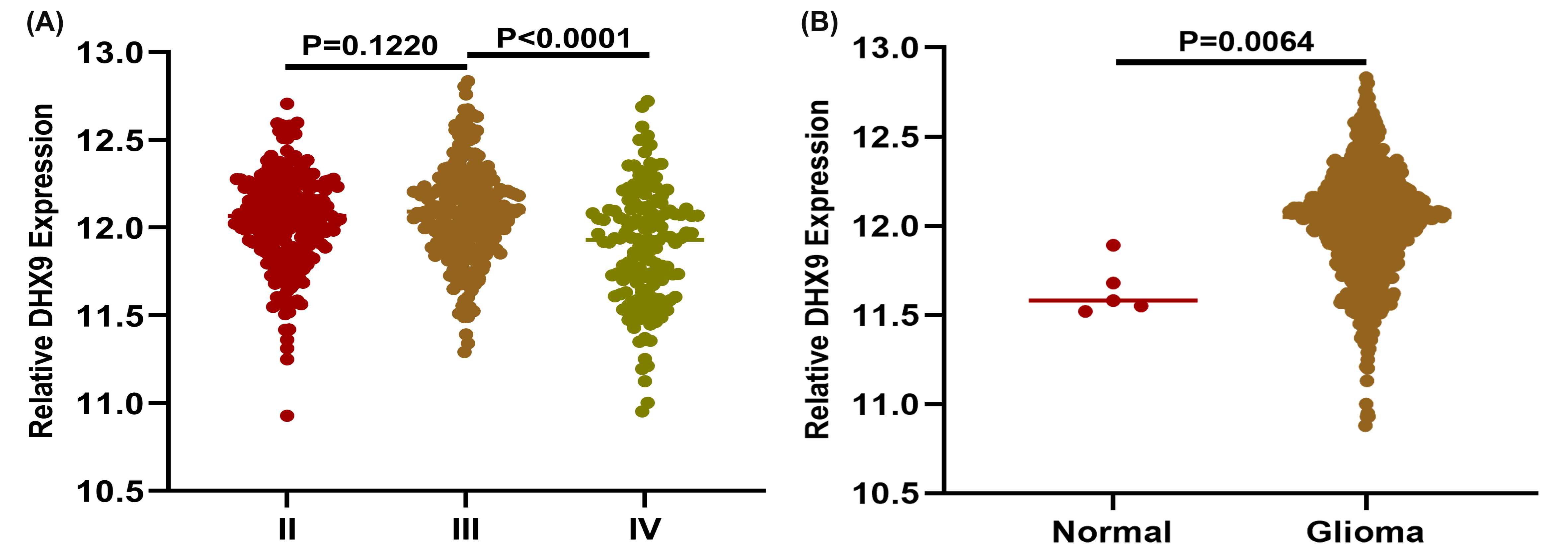

Supplement: Supplementary file 1 — Figure S1 [file CNS-29-988-s001.tif]
